# Electroacupuncture regulates lipid metabolism through PPAR*γ*-associated LXR*α* signaling: from nuclear receptor signaling to cardiovascular protection

**DOI:** 10.3389/fcvm.2026.1997697

**Published:** 2026-10-29

**Authors:** Shuyan Zhang, Jihao Yang, Xinhao Yan, Haichun Zhou

**Affiliations:** 1 College of Acupuncture, Heilongjiang University of Chinese Medicine, Harbin, China; 2 School of Acupuncture and Tuina, Guizhou University of Traditional Chinese Medicine, Guiyang, China; 3 Department of Acupuncture, The Fourth Affiliated Hospital of Heilongjiang University of Chinese Medicine, Harbin, China

**Keywords:** cardiovascular protection, cholesterol efflux, electroacupuncture, lipid metabolism, liver X receptor *α*, PPAR*γ*

## Abstract

Hyperlipidemia is an independent risk factor for atherosclerotic cardiovascular disease, and a substantial proportion of patients remain inadequately controlled on statin therapy because single-target interventions cannot fully reverse the systemic inflammation and tissue injury driven by dysregulated lipid metabolism. Electroacupuncture (EA), a modern refinement of traditional acupuncture,has been reported to lower serum total cholesterol, triglycerides, and low-density lipoprotein cholesterol in clinical trials, yet the molecular basis of these effects has not been systematically synthesized. Here, we review the evidence that EA regulates lipid homeostasis through the peroxisome proliferator-activated receptor *γ* (PPAR*γ*)/liver X receptor *α* (LXR*α*) axis. We first outline the structure and function of these nuclear receptors and their transcriptional crosstalk, and then examine how EA modulates their expression, activity, and co-regulators. We next trace the upstream signal transduction from somatic afferent activation through the neuro–endocrine–immune network, and the downstream effector programs, including cholesterol efflux, fatty-acid oxidation, macrophage M2 polarization, and organ-specific cardiovascular protection. Pharmacological antagonism, PPAR*γ* knockdown, and MerTK deficiency provide causal evidence that the PPAR*γ*-associated efferocytic program contributes to the lipid-lowering and efferocytic effects of EA, whereas the involvement of LXR*α* is supported primarily by its coordinated expression with PPAR*γ* and downstream cholesterol-efflux signaling. We conclude by discussing the heterogeneity of EA parameters, the animal-to-human translational gap, and the limitations of causal inference in the current literature, and outline future directions, including optogenetic dissection of acupuncture neural circuits and PPAR*γ*/LXR*α*-guided standardization of EA protocols.

## 1 Introduction

Hyperlipidemia is an established independent risk factor for atherosclerotic cardiovascular disease, linking dyslipidemia to myocardial infarction, ischemic stroke, and increased cardiovascular mortality (1, 2). The disorder is not merely a passive accumulation of lipids in the vessel wall; dyslipidemia initiates systemic low-grade inflammation and endothelial dysfunction that propagate across metabolic organs. Statins, the first-line pharmacotherapy, lower cholesterol by inhibiting 3-hydroxy-3-methylglutaryl coenzyme A reductase, but a substantial proportion of patients fail to reach lipid targets or discontinue treatment because of resistance, myopathy, and other adverse events (3). Moreover, statin monotherapy does not fully restore the anti-atherogenic program of reverse cholesterol transport (RCT) or resolve the chronic inflammatory milieu that accompanies dyslipidemia (2). These limitations have motivated the search for interventions that simultaneously modulate lipid synthesis, oxidation, and efflux (4).

**Figure 1:**
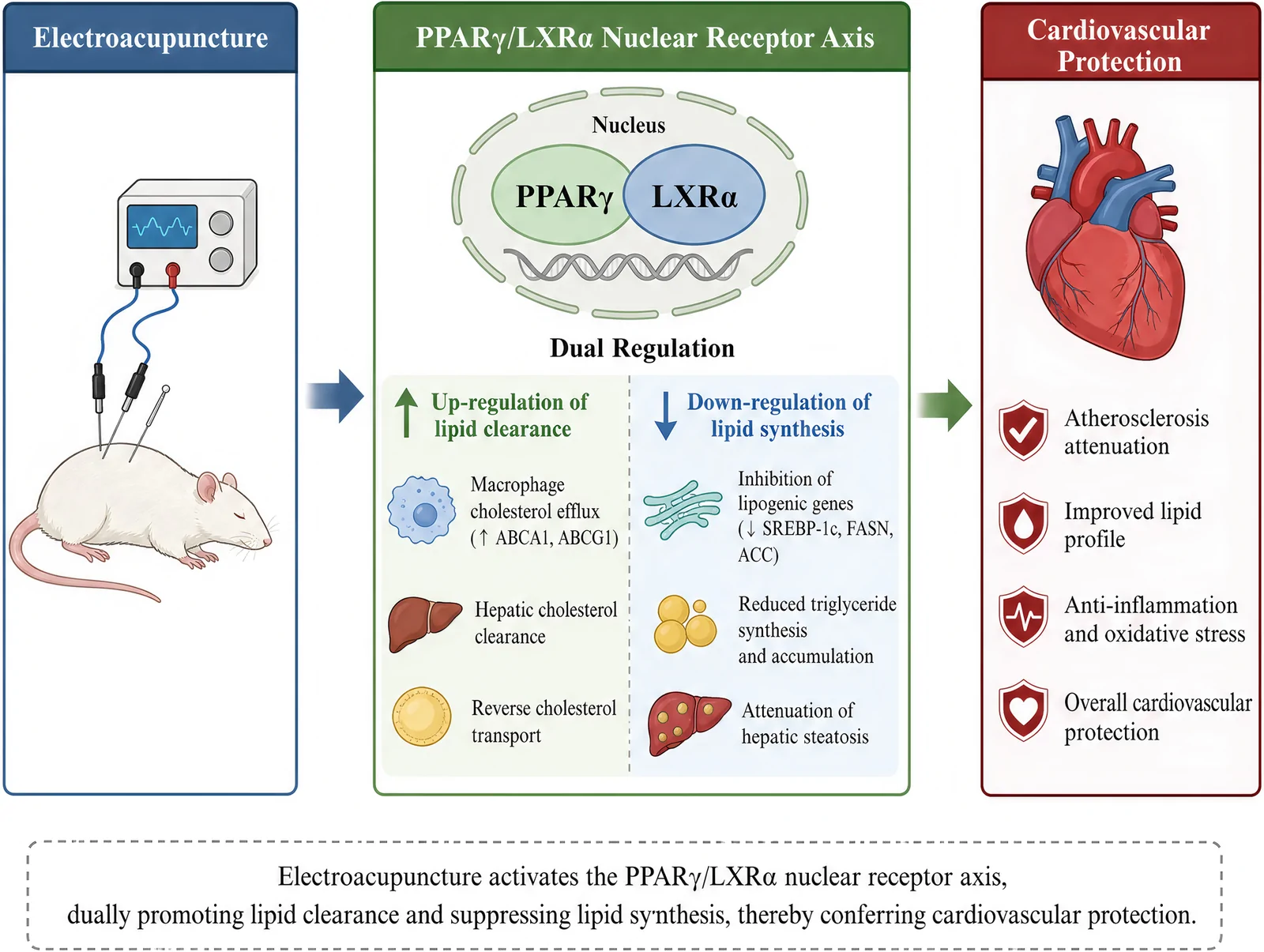
Schematic of the proposed mechanism by which electroacupuncture (EA) confers cardiovascular protection through the pPAR*γ*/lXR*α* nuclear receptor axis. EA activates PPAR*γ* and LXR*α* in the nucleus, which exert dual regulation of lipid metabolism: upregulation of lipid clearance (macrophage cholesterol efflux via ABCA1 and ABCG1, hepatic cholesterol clearance, and reverse cholesterol transport) and downregulation of lipid synthesis (inhibition of lipogenic genes SREBP-1c, FASN, and ACC; reduced triglyceride synthesis and accumulation; attenuation of hepatic steatosis). These effects translate into cardiovascular protection, including attenuation of atherosclerosis, an improved lipid profile, anti-inflammatory and anti-oxidative effects, and overall cardiovascular protection.

**Figure 2:**
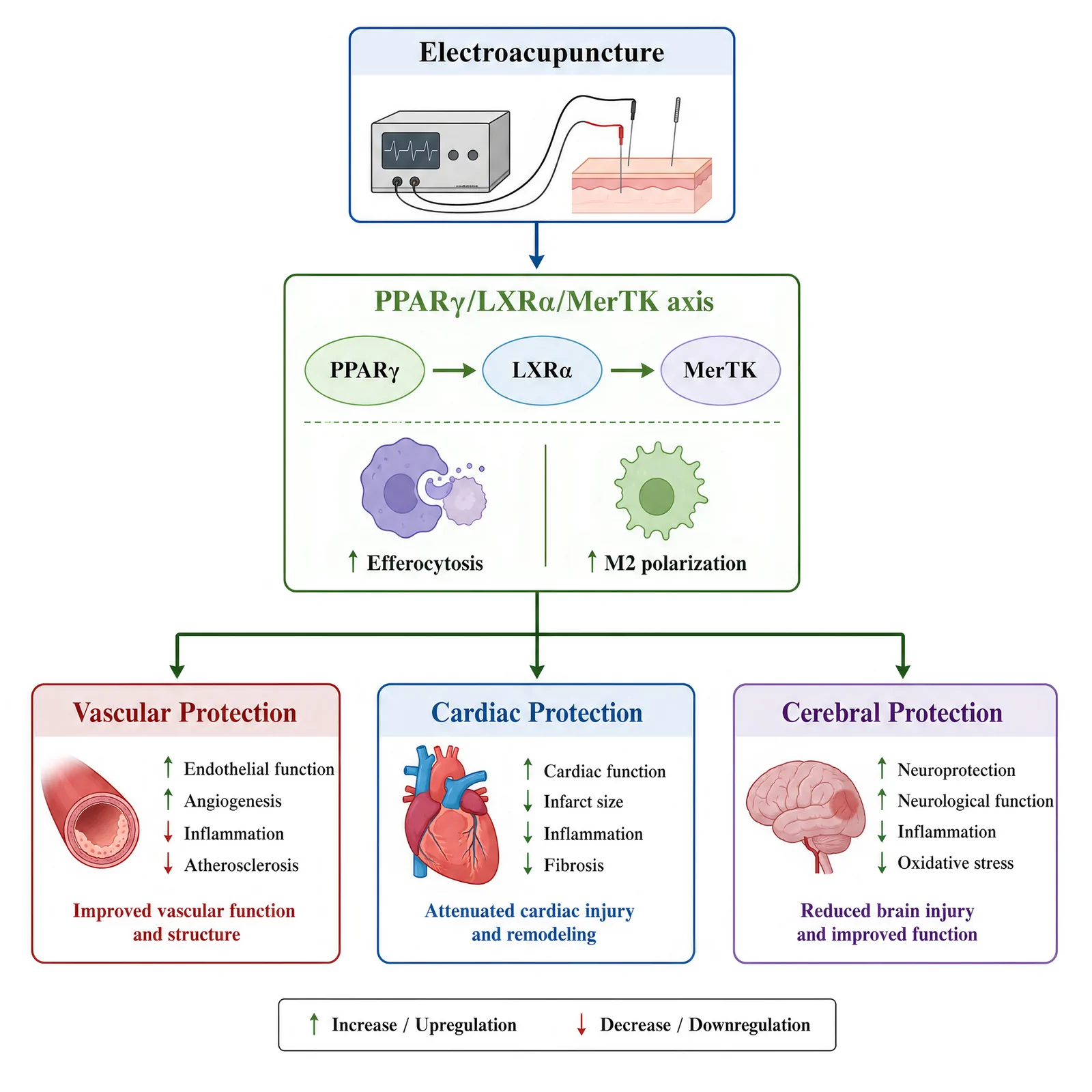
Proposed organ-specific cardiovascular protection mediated by the pPAR*γ*/lXR*α*/merTK axis after EA. Activation of PPAR*γ*, LXR*α*, and MerTK promotes efferocytosis and M2 macrophage polarization, which in turn confer vascular protection (improved endothelial function and angiogenesis; reduced inflammation and atherosclerosis), cardiac protection (improved cardiac function; reduced infarct size, inflammation, and fibrosis), and cerebral protection (neuroprotection and improved neurological function; reduced inflammation and oxidative stress). Upward and downward arrows denote upregulation/increase and downregulation/decrease, respectively.

**Table 1:** Preclinical evidence for electroacupuncture (EA)-associated modulation of pPAR*γ*/lXR*α* signaling.

Model	EA protocol (acupoints)	Molecular changes in the PPAR*γ*/LXR*α* pathway	Functional outcomes	Ref.	LXRα directly measured?
Rat cerebral ischemia/reperfusion	LI11, ST36; 30 min/day, 7 days	↑ PPARγ protein; T0070907 reverses protection	↓ Infarct volume; improved neurological score	(39)	No (PPARγ only)
Rat Alzheimer's disease	GV20, BL23	↑ PPARγ in hippocampal CA1	↓ Pathological protein accumulation	(40)	No (PPARγ only)
Spontaneously hypertensive rat	LR3 (Taichong)	↑ PPARγ in RVLM; GW9662 blocks, agonist mimics	↓ Blood pressure and sympathetic activity	(41)	No (PPARγ only)
HFD-induced hyperlipidemic rat	ST40	↑ PPARγ–LXRα–MerTK, ABCA1, ABCG1 in cardiac macrophages; normalization of lipid-driven PPARγ/LXRα overload in heart and liver	↓ TC, TG, LDL-C; ↑ HDL-C; ↑ cholesterol efflux and efferocytosis; ↓ ectopic lipid deposition	(12)	Yes
HFD-induced hyperlipidemic rat (PPARγ knockdown/MerTK−/−)	ST40	PPARγ knockdown abolishes lipid-lowering and efferocytic effects; MerTK deficiency blocks efferocytosis	EA-enhanced efferocytosis and cholesterol efflux abolished	(12)	No
Rat myocardial ischemia/reperfusion	PC6; 30 min/day, 3 days	↑ PPARγ, IL-10, TGF-*β*, Arg-1; ↓ IL-6, TNF-α; PPARγ inhibitor reverses	↑ Ejection fraction; ↓ myocardial injury and inflammation	(51)	No (PPARγ only)
Rat cerebral ischemia (stroke)	ST36, LI11, GV20	↑ STAT6/PPARγ; microglial M2 polarization	↓ Infarct volume; improved neurological function	(52)	No (PPARγ only)

EA, electroacupuncture; PPARγ, peroxisome proliferator-activated receptor γ; LXRα, liver X receptor α; ABCA1/ABCG1, ATP-binding cassette transporters A1 and G1; SREBP-2, sterol regulatory element-binding protein 2; STAT6, signal transducer and activator of transcription 6; MerTK, MER proto-oncogene tyrosine kinase; TGF-β, transforming growth factor β; Arg-1, arginase 1; IL-6, interleukin 6; IL-10, interleukin 10; TNF-α, tumor necrosis factor α; iNOS, inducible nitric oxide synthase; CD206, mannose receptor; RVLM, rostral ventrolateral medulla; HFD, high-fat diet; TC, total cholesterol; TG, triglycerides; LDL-C, low-density lipoprotein cholesterol; HDL-C, high-density lipoprotein cholesterol; T0070907 and GW9662, PPARγ antagonists.

**Table 2:** Clinical evidence for acupuncture/electroacupuncture (EA) in lipid metabolism.

Study design/population	Intervention	Key lipid findings	Ref.
Network meta-analysis; obesity with insulin resistance (14 RCTs)	Acupuncture vs. control	TC −1.82 (−3.36 to −0.28); LDL-C −1.37 (−2.09 to −0.66); HDL-C + 0.87 (0.52–1.22) mmol/L	(9)
Meta-analysis; dyslipidemia	Acupuncture + statin vs. statin	Greater reduction in TC and LDL-C; fewer adverse events	(3)
Systematic review and meta-analysis; coronary heart disease	Acupuncture-related treatments	Significant improvement in blood lipid levels	(1)
Systematic review and meta-analysis; NAFLD	Acupoint therapy	Improved liver enzymes and lipid profile	(7, 97)
Clinical study; NAFLD with hyperlipidemia	EA at ST40	Improved serum lipid profile	(10)
Systematic review and meta-analysis; coronary heart disease	Acupuncture	Improved glycolipid metabolism	(8)
Network meta-analysis; obese-type polycystic ovary syndrome	Acupuncture/moxibustion	Improved glycolipid metabolism	(98)
Prospective cohort; knee osteoarthritis	Acupuncture	Reversed disordered serum lipidomic species	(11)

TC, total cholesterol; TG, triglycerides; LDL-C, low-density lipoprotein cholesterol; HDL-C, high-density lipoprotein cholesterol; NAFLD, non-alcoholic fatty liver disease; RCT, randomized controlled trial.

Electroacupuncture (EA), a modern refinement of traditional acupuncture that delivers low-frequency electrical stimulation through needles inserted at specific acupoints, has shown consistent efficacy across metabolic disorders (5, 6). Multiple randomized controlled trials and meta-analyses have demonstrated that EA lowers serum total cholesterol (TC), triglycerides (TG), and low-density lipoprotein cholesterol (LDL-C) in patients with hyperlipidemia, non-alcoholic fatty liver disease (NAFLD), and type 2 diabetes (7, 8). A network meta-analysis of 14 randomized controlled trials in patients with obesity and insulin resistance reported that acupuncture significantly reduced TC (mean difference −1.82, 95% CI −3.36 to −0.28 mmol/L) and LDL-C (−1.37, 95% CI −2.09 to −0.66 mmol/L) while raising high-density lipoprotein cholesterol (HDL-C) (0.87, 95% CI 0.52–1.22 mmol/L) (9). In clinical practice, EA at ST36 (Zusanli) and ST40 (Fenglong), often combined with conventional treatment, further lowers TG and TC with fewer adverse events than drug monotherapy (10). EA also corrects the disordered serum lipidome in patients with knee osteoarthritis, reversing abnormal levels of phosphatidylethanolamine and lysophosphatidylcholine species (11). These observations position EA as a promising adjunct for lipid management, but they leave a central question unanswered: how does a peripheral electrical stimulus reprogram lipid metabolism at the molecular level?

The molecular mechanisms underlying the lipid-lowering effects of EA remain incompletely defined. Most studies have focused on phenotypic endpoints—weight loss and reduced lipid levels—whereas the conversion of an acupuncture stimulus into intracellular molecular events has remained a “black box”. Emerging evidence implicates the nuclear receptors peroxisome proliferator-activated receptor *γ* (PPAR*γ*) and liver X receptor *α* (LXR*α*) as central mediators of EA's metabolic effects (12). PPAR*γ* governs fatty-acid uptake, storage, and macrophage polarization, whereas LXR*α*, the master sensor of cellular cholesterol, drives RCT (13). Whether EA engages these receptors directly or acts indirectly through upstream neuro–endocrine–immune circuits remains unresolved: some studies emphasize AMPK/ACC-dependent glucolipid regulation (14, 15), whereas others invoke SIRT1/NF-*κ*B signaling (16, 17). The position of the PPAR*γ*/LXR*α* axis within this multi-target network has not been systematically integrated. In this review, we synthesize the evidence linking EA to PPAR*γ*/LXR*α*-dependent regulation of lipid metabolism, trace the signal cascade from peripheral stimulation to transcriptional control and organ protection, and evaluate the strength of the causal evidence. Our aim is to provide a biological framework for the clinically established lipid-lowering effects of EA (18).

For this narrative review, we conducted a structured literature search in PubMed, Web of Science, Embase, and the Cochrane Library, supplemented by manual screening of reference lists, for studies published up to October 2026. Search terms combined electroacupuncture or acupuncture with PPAR*γ*, liver X receptor, lipid metabolism, cholesterol efflux, reverse cholesterol transport, and cardiovascular protection. Clinical evidence was drawn preferentially from randomized controlled trials, meta-analyses, and systematic reviews, whereas mechanistic evidence was drawn from original animal and cellular studies and authoritative reviews of nuclear-receptor biology. Studies were excluded if they were duplicates, lacked full text, or were unrelated to the EA–PPAR*γ*/LXR*α* axis.

## 2 The PPAR*γ*/LXR*α* axis: molecular framework of lipid homeostasis

PPAR*γ* and LXR*α* belong to the nuclear receptor superfamily and act as ligand-activated transcription factors that maintain lipid homeostasis. PPAR*γ* regulates triglyceride metabolism, lipid uptake, cholesterol efflux, and macrophage polarization. LXR*α* (NR1H3) and its isoform LXR*β* (NR1H2) function as cholesterol sensors; LXR*α* is enriched in metabolically active tissues such as the liver, whereas LXR*β* is more broadly expressed (19). By sensing intracellular lipid levels, these receptors coordinate the expression of downstream genes to sustain systemic lipid balance and to modulate inflammatory responses (20). Their function extends beyond the liver and macrophages: LXR*α* regulates steroidogenesis and reproduction (21), modulates Kupffer cells and natural killer T cells in steatohepatitis (22), and its environmental modulation by endocrine-disrupting compounds perturbs hepatic lipid metabolism (23).

### 2.1 PPAR*Γ*/RXR heterodimer and transcriptional control

PPAR*γ* must heterodimerize with the retinoid X receptor (RXR) to bind PPAR response elements in the promoters of target genes, where the recruitment of coactivators or corepressors dictates transcriptional activation or repression (24). PPAR*γ* activity is regulated not only by ligand availability but also by post-translational modification and the balance of co-regulatory proteins. Notably, PPAR*γ* activation directly increases LXR*α* expression; PPAR*γ* agonists such as rosiglitazone upregulate LXR*α* and its downstream efflux transporters, establishing a transcriptional hierarchy in which PPAR*γ* operates upstream of LXR*α* (25–27). The stability of PPAR*γ* itself is also functionally relevant, because compounds that reduce its ubiquitin-dependent degradation enhance its transcriptional output (28). Through these mechanisms, the PPAR*γ*/RXR heterodimer promotes triglyceride storage in adipose tissue while coordinating lipid oxidation and transport in the liver and macrophages.

### 2.2 LXR*α* and reverse cholesterol transport

LXR*α* functions as an intracellular cholesterol sensor that is activated by oxysterols such as 27-hydroxycholesterol. Upon activation, LXR*α* directly upregulates the ATP-binding cassette transporters ABCA1 and ABCG1, promoting the efflux of free cholesterol from macrophages to apolipoprotein A-I and HDL and thereby preventing foam-cell formation (29). The two LXR isoforms exert common yet distinct transcriptional actions in macrophages (30), and LXR*α* also regulates aquaglyceroporins and membrane trafficking in metabolic organs (31). LXR*α* limits cholesterol influx by inducing the E3 ubiquitin ligase IDOL and represses hepatic cholesterol synthesis through the long non-coding RNA LeXis, providing integrated control of cholesterol uptake, transport, efflux, and synthesis (32). These actions are critical for reducing atherosclerotic plaque burden. Beyond the cardiovascular system, LXR signaling has been implicated in inflammation-driven tumorigenesis, including inflammatory bowel disease and breast cancer (33, 34). However, LXR*α* activation is tissue-dependent; in the liver it can simultaneously induce SREBP-1c and promote lipogenesis, a property that has limited the development of LXR agonists as systemic therapeutics.

### 2.3 PPAR*γ*–LXR*α* crosstalk

PPAR*γ* and LXR*α* engage in bidirectional crosstalk that positions them as a coherent regulator*y* axis of lipid homeostasis. Convergent evidence indicates that PPAR*γ* acts upstream of LXR*α*: PPAR*γ* activation upregulates LXR*α* and its downstream targets ABCA1/ABCG1, whereas pharmacological inhibition of PPAR*γ* with GW9662 or PPAR*γ* knockdown abolishes the LXR*α*/ABCA1 induction and cholesterol efflux triggered by PPAR*γ* agonists or natural products. The two receptors also share genomic regulatory regions and coordinately regulate genes involved in cholesterol and fatty-acid metabolism, and this axis mediates phenotypic plasticity in other pathological settings such as pulmonary fibrosis (35). The interplay extends to signaling integration, as exemplified by the AMPK–PPAR*γ*–LXR*α* cascade, which promotes cholesterol efflux and suppresses inflammation (36, 37). At the immune level, PPAR*γ* and LXR*α* cooperate to restrain inflammatory cytokine release and to drive M2 macrophage polarization, effects that are directly relevant to the anti-inflammatory actions of EA (38).

## 3 Electroacupuncture modulates PPAR*γ*-associated LXR*α* signaling

A growing body of preclinical evidence indicates that EA can modulate PPAR*γ* signaling and is accompanied by changes in LXR*α*-associated lipid metabolic pathways (39). However, the available evidence does not yet establish a uniform causal sequence from acupoint stimulation to neural circuit activation, followed by tissue-specific regulation of PPAR*γ*/LXR*α* and subsequent lipid metabolic outcomes. In particular, most EA studies have examined PPAR*γ* expression or activity, whereas direct manipulation of LXR*α* in EA-treated models remains limited. Throughout this review we distinguish three levels of evidence: (i) direct causal evidence, obtained in EA experiments combined with loss-of-function or pharmacological validation (PPAR*γ* antagonists, small interfering RNA, or gene knockout); (ii) direct but correlational evidence, from EA experiments without genetic or pharmacological perturbation; and (iii) indirect evidence, from non-EA interventions (pharmacological agents, natural products, or related mechanistic models) that engage the same molecular pathway and are used to infer candidate mechanisms that EA may share.

From this perspective, the current evidence supports PPAR*γ* as a relatively well-characterized molecular target of EA in several experimental models, whereas the involvement of LXR*α* is better interpreted as a coordinated or downstream component of PPAR*γ*-associated lipid metabolic regulation. The neural and autonomic mechanisms linking peripheral electroacupuncture stimulation to these nuclear receptor pathways remain insufficiently characterized. Thus, the proposed EA–PPAR*γ*/LXR*α* relationship should be regarded as an evidence-supported molecular framework rather than a fully established linear neural-to-nuclear signaling cascade.

### 3.1 EA upregulates PPAR*γ* expression and activity

In models of cerebral ischemia/reperfusion injury (CIRI), EA at LI11 (Quchi) and ST36 delivered for 30 min daily over 7 days reduced infarct volume and neurological deficits while increasing PPAR*γ* protein expression in brain tissue; the protective effects were reversed by the PPAR*γ* antagonist T0070907, indicating that PPAR*γ* mediates the neuroprotective action of EA (39). In a rat model of Alzheimer's disease, EA at GV20 (Baihui) and BL23 (Shenshu) similarly upregulated PPAR*γ* expression in the hippocampal CA1 region and reduced the accumulation of pathological proteins (40). In the cardiovascular system, EA at LR3 (Taichong) in spontaneously hypertensive rats increased PPAR*γ* protein in the rostral ventrolateral medulla (RVLM), and this expression correlated inversely with blood pressure and sympathetic vasomotor activity; microinjection of the PPAR*γ* inhibitor GW9662 abolished the antihypertensive effect, whereas a PPAR*γ* agonist mimicked it (41). Collectively, these studies establish that EA engages PPAR*γ* in a tissue-specific manner and that PPAR*γ* is required for the protective effects of EA across ischemic, neurodegenerative, and hypertensive contexts.

EA regulation of PPAR*γ* is context- and cell-type-dependent. In high-fat diet (HFD)-induced hyperlipidemic rats, EA at ST40 lowered serum lipids and reduced ectopic lipid deposition in the heart and liver, an effect accompanied by normalization of the lipid-driven elevation of PPAR*γ* and LXR*α* protein in these tissues (12). In parallel, EA enhanced reverse cholesterol transport and macrophage efferocytosis by upregulating the PPAR*γ*–LXR*α*–MerTK pathway, including ABCA1, ABCG1, PPAR*γ*, LXR, and MerTK, specifically in cardiac macrophages. Both the lipid-lowering and efferocytic effects were abolished by PPAR*γ* knockdown, and the efferocytic effect was blocked in MerTK-deficient rats, establishing the PPAR*γ*–LXR*α*–MerTK axis as the effector pathway (12). Thus, EA simultaneously normalizes the pathological nuclear-receptor overload imposed by hyperlipidemia and activates the functional cholesterol-efflux and efferocytosis program in macrophages—an apparent paradox that underscores the cell-type specificity of EA's actions. EA may also modulate lymphatic endothelial markers and PPAR*γ*-related genes in the primary vascular system, further contributing to vascular homeostasis (42).

Throughout this review, the term PPAR*γ*/LXR*α* axis denotes the coordinated nuclear-receptor regulation of lipid metabolism, cholesterol efflux, and inflammatory resolution, whereas the PPAR*γ*–LXR*α*–MerTK pathway refers to the specific downstream effector program through which this axis promotes macrophage cholesterol efflux and efferocytosis; MerTK therefore operates as a downstream effector of the axis rather than as an equivalent term.

Although this apparent paradox has been attributed to cell-type specificity, a deeper reading indicates that the outcome of PPAR*γ*/LXR*α* activation is governed by the tissue context and the prevailing metabolic state rather than by a fixed direction of signaling (43). In the heart and liver of HFD-fed rats, hyperlipidemia itself drives an abnormal elevation (overload) of PPAR*γ* and LXR*α* protein, and the EA-mediated normalization of this pathological overactivation is plausibly protective because it restrains maladaptive lipid signaling while preserving homeostatic tone. By contrast, in cardiac macrophages, the same receptors engage the PPAR*γ*–LXR*α*–MerTK effector program to promote cholesterol efflux and efferocytosis, which is beneficial (12). These observations indicate that PPAR*γ*/LXR*α* signaling can exert distinct, sometimes opposing, effects in different cell types and disease contexts—protective in macrophages engaged in cholesterol clearance, yet capable of promoting lipogenic programs in hepatocytes (44)—and that the balance between benefit and harm depends on the cellular context, the duration and intensity of receptor activation, and the coexisting metabolic state (43, 44).

Importantly, the available evidence provides stronger support for the downstream role of PPAR*γ* than for the upstream mechanism by which EA regulates PPAR*γ*. In cerebral ischemia/reperfusion models, EA-induced enhancement of PPAR*γ* signaling was attenuated by pharmacological antagonism, supporting a causal contribution of PPAR*γ* to the observed tissue-protective effects. However, these studies did not determine whether PPAR*γ* was regulated through a defined central neural circuit, autonomic pathway, endocrine signal, or a combination of these mechanisms. Therefore, the evidence currently supports an EA–PPAR*γ* association with functional consequences, but does not establish a specific neural relay linking acupoint stimulation to PPAR*γ* regulation.

### 3.2 EA-mediated LXR*α* signaling

Direct studies of EA and LXR*α* as a single target remain limited, but multiple lines of evidence indicate that EA regulates LXR*α* through its tight transcriptional coupling to PPAR*γ* (26). In hyperlipidemic rats, EA influenced LXR*α* expression in parallel with PPAR*γ* and modulated the downstream cholesterol-efflux transporters ABCA1 and ABCG1, thereby promoting reverse cholesterol transport (12). This pattern mirrors the effects of PPAR*γ*/LXR*α*-activating pharmacological agents and natural products, in which PPAR*γ* activation is consistently accompanied by LXR*α* upregulation and ABCA1/ABCG1-dependent cholesterol efflux. EA may also activate LXR*α* indirectly through upstream neuro–endocrine signals: studies have implicated SIRT1/NF-*κ*B-dependent improvement of hepatic lipid metabolism and USP20/mTOR/HMGCR-mediated suppression of hepatic cholesterol synthesis. Because PPAR*γ* can directly regulate LXR*α* promoter activity, EA-mediated modulation of PPAR*γ* is likely to extend to LXR*α* through crosstalk. LXR*α* activation suppresses inflammation and promotes M2 macrophage polarization, consistent with the anti-inflammatory and anti-atherosclerotic phenotypes observed after EA (38). The LXR*α* branch of EA's mechanism therefore appears to operate as a multi-layered network involving PPAR*γ*-coordinated activation, upregulation of downstream transporters, and suppression of inflammation; the precise molecular details await validation in receptor-specific knockout models (13).

### 3.3 Coregulators in EA action

Nuclear-receptor transcriptional activity depends on the recruitment and balance of coactivators and corepressors. Direct evidence linking EA to specific co-regulators is scarce; the candidate mechanisms discussed below are therefore drawn from indirect evidence in non-EA models. The coactivator SRC-1 mediates LXR*α*-dependent transcription of GLUT4 during adipogenesis and glucose metabolism (45). The chromatin-remodeling factor HMGB1 represses LXR*α* and PPAR*γ* activity by occupying their regulatory regions under nutritional stress, protecting mice from fatty-liver progression; HMGB1 deficiency enhances LXR*α* and PPAR*γ* activity (46, 47). These findings raise the possibility that EA alters the DNA-binding or complex-assembly properties of PPAR*γ*/LXR*α* by modulating epigenetic or co-regulatory factors analogous to HMGB1. In other non-EA interventions, plant extracts enhance PPAR*γ* stability by reducing its ubiquitination, and microRNAs such as miR-150 and miR-7683-3p modulate PPAR*γ*/LXR*α* signaling by targeting AdipoR2 or HOXA1 (48, 49), while miR-149-5p regulates adipocyte differentiation downstream of these receptors (50). Given its multi-target nature, EA may influence co-regulator activity through changes in cellular redox state, kinase phosphorylation such as AMPK (36), or microRNA profiles, thereby optimizing the transcriptional efficiency of the PPAR*γ*/LXR*α* heterodimer. Future studies should determine whether EA specifically recruits coactivators such as SRC-1 and PGC-1*α* or relieves repression by corepressors such as NCoR and SMRT.

## 4 From peripheral stimulation to nuclear receptor activation

### 4.1 The neuro–endocrine–immune network transduces EA signals

The lipid-regulating effects of EA begin with the reception of the electrical stimulus by peripheral nerve endings and are propagated systemically through the neuro–endocrine–immune network (6, 53). EA at acupoints such as ST36 and PC6 (Neiguan) activates the vagus-mediated cholinergic anti-inflammatory pathway (CAP), influencing *α*7 nicotinic acetylcholine receptor (*α*7nAChR)-dependent NF-*κ*B/NLRP3 signaling and modulating metabolic pathways including AMPK–SIRT1–PGC-1*α* (53). In obese mice, EA inhibits the migration of intestinal dendritic cells to mesenteric adipose tissue, reduces CCR7 expression, and decreases inflammatory-cell infiltration in adipose tissue (54). EA also enhances satiety signaling and promotes white-adipose-tissue browning through the modulation of hypothalamic nuclei and sympathetic–immune interactions (5), and gut-microbiota remodeling has been proposed as an additional mediator of its metabolic effects (55). In a myocardial ischemia/reperfusion (MIRI) model, EA at PC6 activated the PPAR*γ*/NF-*κ*B pathway, implicating the transmission of neural signals to cardiac immune cells and consequent changes in macrophage phenotype (51). Metabolic imaging with 18F-FDG/PET further demonstrates EA-induced changes in glucose utilization and AMPK*α* phosphorylation after ischemic stroke (56). These observations delineate a transduction network in which the acupuncture signal travels from peripheral somatic afferents to central or regional ganglia and then, through neurotransmitter and hormonal release, acts on immune cells and metabolic organs.

### 4.2 Ppar*γ*/LXR*α*, inflammation, and macrophage polarization

PPAR*γ* and LXR*α* are not only metabolic transcription factors but also hubs that link metabolism to immunity; their activation state in myeloid cells shapes both lipid handling and inflammatory disease outcomes (57). Activation of the PPAR*γ*/LXR*α* pathway promotes anti-inflammatory M2 polarization (38). In a rat MIRI model, EA increased myocardial expression of PPAR*γ*, IL-10, TGF-*β*, Arg-1, and CD163 (M2 markers) while decreasing IL-6, TNF-*α*, iNOS, and CD86 (M1 markers); PPAR*γ* inhibition reversed this protective effect (51). In cerebral ischemia, EA promoted microglial M2 polarization through the assembly of a STAT6/PPAR*γ* transcriptional complex, conferring neuroprotection (52, 58). In obese mice, EA reduced pro-inflammatory TNF-*α* and iNOS in adipose tissue and increased the anti-inflammatory cytokines IL-4 and IL-10 and the M2 marker CD206 (59). Conversely, in non-EA experimental settings, suppression of the PPAR*γ*/LXR*α* pathway exacerbates inflammation: IL-32*α* dose-dependently reduces PPAR*γ*, LXR*α*, and ABCA1 expression, impairing cholesterol efflux and promoting a pro-inflammatory environment (60), and TLR4 activation aggravates vascular inflammation and lipid accumulation by inhibiting PPAR*γ*/LXR*α* expression (61). By upregulating PPAR*γ*/LXR*α*, EA thus reshapes the balance of macrophage polarization, suppresses pro-inflammatory NF-*κ*B signaling, and establishes an anti-inflammatory microenvironment in systemic and local tissues.

### 4.3 Lipid metabolic reprogramming: fatty-acid oxidation and cholesterol efflux

Under the control of the PPAR*γ*/LXR*α* pathway, EA-induced lipid metabolic reprogramming manifests as enhanced fatty-acid oxidation and activation of reverse cholesterol transport (36). As the upstream regulator of LXR*α*, PPAR*γ* cooperates with LXR*α* to upregulate ABCA1 and ABCG1, the key steps in macrophage cholesterol efflux. In HFD-fed rats, EA upregulated ABCA1, ABCG1, and MerTK protein in myocardial macrophages, promoting reverse cholesterol transport and efferocytosis in a manner dependent on the PPAR*γ*–LXR*α*–MerTK pathway (12). In atherosclerotic models, pharmacological and natural-product activation of the PPAR*γ*–LXR*α*–ABCA1/G1 pathway—indirect evidence from non-EA interventions—promotes cholesterol efflux from foam cells and inhibits plaque formation (62–68). On the fatty-acid side, EA may promote lipolysis by regulating relevant gene expression; studies suggest that EA inhibits lipid synthesis through the AMPK/ACC pathway (14, 15), and PPAR*γ* activation is closely associated with fatty-acid oxidation. In co-cultured primary hepatocytes and macrophages, PPAR*γ* upregulation increases fatty-acid oxidation and reduces reactive oxygen-species production (69). Dietary factors and natural products modulate PPAR signaling and lipid metabolism in parallel (70, 71). EA also improves the hepatic lipid metabolome, reversing abnormal levels of phospholipids, lysophospholipids, and triglycerides (72). Through the PPAR*γ*/LXR*α* axis, EA therefore drives ABCA1/ABCG1-mediated cholesterol efflux to clear vascular lipid deposits while balancing fatty-acid oxidation and synthesis to restore lipid homeostasis.

## 5 From lipid metabolism to cardiovascular protection

### 5.1 Anti-atherosclerosis and vascular protection

EA attenuates the progression of atherosclerosis by promoting reverse cholesterol transport and enhancing macrophage efferocytosis. In HFD-induced hyperlipidemic rats, EA reduced serum TC, TG, and LDL-C, upregulated ABCA1, ABCG1, PPAR*γ*, LXR*α*, and MerTK in myocardial macrophages, promoted lipid efflux, and alleviated lipid deposition in the myocardium and liver (12). PPAR*γ* knockdown or MerTK deficiency abolished these effects, confirming the causal role of the PPAR*γ*–LXR*α*–MerTK pathway (12). EA also protects vascular endothelial function. In rats with hyperlipidemia complicated by cerebral ischemia, EA preconditioning at ST40 reduced lipid levels while attenuating glial fibrillary acidic protein immunoreactivity and enhancing nerve growth-factor expression in the hippocampus, suggesting integrated protection of the vascular and neural microenvironment (73). Although direct molecular markers of plaque stability such as MMP-9 have been reported mainly in phytotherapy studies (74, 75), the PPAR*γ*/LXR*α*-dependent optimization of the lipid profile reduces lipid deposition in the vessel wall at its source and thereby provides the metabolic basis for delaying atherosclerotic progression (2).

### 5.2 Myocardial protection against ischemia/reperfusion injury and oxidative stress

Both EA preconditioning and post-treatment confer myocardial protection, mediated by PPAR*γ*-dependent anti-inflammatory actions and Nrf2/HO-1-dependent antioxidant responses (76). In MIRI models, EA at PC6 improved cardiac ejection fraction and reduced myocardial fiber damage and inflammatory infiltration; mechanistically, EA upregulated myocardial PPAR*γ*, IL-10, TGF-*β*, and Arg-1, promoted M2 macrophage polarization, and suppressed NF-*κ*B p65 phosphorylation and the pro-inflammatory cytokines IL-6 and TNF-*α* (51). These cardioprotective and anti-inflammatory effects were reversed by PPAR*γ* inhibition (51). With respect to oxidative stress, EA preconditioning reduced myocardial reactive oxygen species (ROS) and malondialdehyde (MDA) while increasing superoxide dismutase (SOD) and heme oxygenase-1 (HO-1) expression; this protection depended on Nrf2 activation, because the Nrf2 inhibitor ML385 abolished it (77). EA further protects the myocardium by modulating mitochondrial function and cell-death pathways—for example, by activating the XBP1/GRP78/Akt pathway to suppress cardiomyocyte apoptosis (78) or by downregulating miR-451 to enhance AMPK signaling, thereby reducing infarct size and apoptosis (79).

### 5.3 Cerebral protection: blood–brain-barrier integrity and neuroinflammation

EA exerts neuroprotective effects in cerebral ischemia/reperfusion injury (CIRI), primarily by suppressing neuroinflammation, reducing oxidative stress, and preserving blood–brain-barrier (BBB) integrity (80). Across multiple CIRI models, EA at ST36, LI11, or GV20 reduced infarct volume and improved neurological scores (81). The anti-inflammatory mechanism involves STAT6/PPAR*γ* signaling: EA promotes microglial M2 polarization, increases anti-inflammatory cytokine release, and inhibits M1 polarization and NF-*κ*B activity; STAT6 phosphorylation inhibition attenuated these effects (52), and the PPAR*γ* antagonist T0070907 reversed the suppression of oxidative stress and neuronal death after cerebral ischemia (39). For BBB protection, EA maintains structural integrity by upregulating tight-junction proteins such as ZO-1 and occludin (82), and may indirectly reduce cerebral inflammation by regulating intestinal barrier function and the Treg/*γδ* T-cell balance (83). EA also inhibits ferroptosis in the ischemic penumbra through GSK-3*β*/Nrf2 signaling (84), suppression of the ferroptosis-related protein ZIP14 (85), and AMPK pathway modulation (86), thereby attenuating oxidative damage and cell death through multiple routes.

## 6 Evidence integration: model systems and causal validation

### 6.1 Rodent models confirm EA-mediated lipid lowering via PPAR*γ*/LXR*α*

Rodent models provide convergent evidence that EA regulates lipid metabolism through the PPAR*γ*/LXR*α* pathway. In HFD-induced hyperlipidemic rats, EA lowered serum TC, TG, and LDL-C while raising HDL-C (15). Mechanistic studies show that EA at ST36 upregulates PPAR*γ* and LXR*α* in hepatic and vascular tissues and promotes the expression of the cholesterol-efflux transporters ABCA1 and ABCG1, accelerating reverse cholesterol transport. In ApoE-knockout atherosclerotic mice, related non-EA interventions upregulate PPAR*γ*, LXR*α*, and ABCA1 mRNA and protein in the aorta and liver, improving lipid deposition and endothelial injury (87). Loss-of-function experiments provide the strongest causal evidence: PPAR*γ*-specific small interfering RNA or antagonists such as GW9662 and T0070907 reverse EA-induced improvements in serum lipids, myocardial macrophage efferocytosis, and neuroinflammation (12, 39, 52), and in spontaneously hypertensive rats, EA lowers sympathetic vasomotor activity by increasing RVLM PPAR*γ*, an effect completely blocked by PPAR*γ* inhibition (41).

### 6.2 Cellular evidence confirms the key molecular targets

Cellular studies further clarify the molecular mechanisms by which EA and related active principles act on the PPAR*γ*/LXR*α* pathway. Except where stated, the cellular experiments below constitute indirect evidence from non-EA models. In HepG2 cells, treatment that increases SIRT1 activity upregulates PPAR*α*, LXR*β*, and PGC-1*α* at both mRNA and protein levels while suppressing the lipogenic factor SREBP-1c (88). In macrophages, apoptotic-cell-induced STAT6 phosphorylation upregulates PPAR*γ*, LXR*α*, and ABCA1, and this effect is reversed by PPAR*γ* knockdown or the antagonist GW9662, confirming the central role of the PPAR*γ*/LXR*α* axis in macrophage lipid efflux (89). In an oleate-induced insulin-resistant HepG2 model, silencing PPAR*α* aggravates lipid deposition and impairs antioxidant capacity (90). Although direct *in vitro* experiments with EA stimulation are scarce, studies of natural products with acupuncture-like effects provide a mechanistic reference: many such compounds bind to key residues of PPAR*γ* and LXR*α* in molecular-docking analyses, activate downstream signaling, and inhibit lipid accumulation (91–96). These observations collectively indicate that the PPAR*γ*/LXR*α* axis is a druggable and EA-responsive hub of lipid regulation.

### 6.3 Clinical evidence and translational status

Clinical evidence supporting the lipid-modulating effects of EA has been summarized in multiple systematic reviews and meta-analyses. In patients with dyslipidemia, acupuncture combined with statins is superior to statin monotherapy in lowering TC and LDL-C, with a favorable safety profile (3). In patients with coronary heart disease, acupuncture-related treatments significantly improved blood lipid levels (1), and in patients with NAFLD, acupoint therapy improved liver enzymes and lipid profiles (97). EA at ST40 in patients with NAFLD and hyperlipidemia improved the serum lipid profile (10). Meta-analyses in obese patients with insulin resistance (9), in obese-type polycystic ovary syndrome (98), and in patients with coronary heart disease (8) confirmed reductions in TC and LDL-C and increases in HDL-C, and acupuncture combined with metformin improved insulin sensitivity in diabetic patients (99). Despite this evidence, the clinical literature remains limited by small sample sizes, heterogeneous EA protocols, and the frequent absence of sham-controlled designs. Moreover, no trial has yet measured PPAR*γ*/LXR*α* expression or its downstream effectors in patient samples; bridging this gap will be essential to translate the preclinical mechanism into validated clinical biomarkers.

In addition, publication bias and small-study effects may inflate the apparent efficacy of acupuncture in the cited meta-analyses, and funnel-plot or sensitivity analyses were rarely reported; these methodological caveats should further temper the strength of the clinical conclusions.

## 7 Controversies, limitations, and technical challenges

### 7.1 Heterogeneity of EA parameters

The biological effects of EA depend critically on stimulation parameters, including frequency, waveform, intensity, and acupoint selection, and the heterogeneity of these parameters across studies impedes the formation of unified mechanistic conclusions. In terms of frequency and waveform, the optimal parameters differ by disease and therapeutic goal. Higher treatment frequency (>3 sessions/week) is more effective for symptom relief in acute stages of degenerative spinal or joint disease, but exhibits a “J-shaped curve” and plateau effects, whereas peripheral neuropathy is insensitive to frequency changes (100). Clinical data mining shows that EA for cancer pain most commonly uses dense-disperse waves at 2/100 Hz (101), whereas treatment for knee osteoarthritis predominantly uses continuous waves at low frequency (mainly 2 Hz) (102). In post-stroke dysphagia, dense-disperse waveforms outperform other waveforms, and sessions of ≥30 min combined with specific waveforms are effective, whereas stimulation frequency alone does not show statistically significant differences (103). With respect to acupoint specificity, forward and reverse inference from clinical data supports the specificity of certain acupoint indications (104, 105), although unclear terminology and the lack of biological grounding limit its evidence-based status (106). Animal experiments provide more direct evidence: in a rabbit model of acute bradycardia, only PC6 improved heart rate and induced local mast-cell degranulation and adenosine release, whereas non-meridian and control points were ineffective (107). Because parameter standards are lacking, results across studies cannot be directly compared; a blinded frequency trial for postoperative musculoskeletal pain noted that most studies lack sham or EA-free control groups, precluding assessment of the absolute or specific efficacy of particular frequencies (e.g., 2 Hz vs. 100 Hz) (108).

To improve comparability and reproducibility, future EA studies should follow the STandards for Reporting Interventions in Clinical Trials of Acupuncture (STRICTA), which specify the transparent reporting of stimulation frequency, waveform, intensity, duration, number and frequency of sessions, needling depth and manipulation, acupoint-selection rationale, and the nature of control or sham interventions (109). Adherence to such structured reporting would help disentangle the parameter-related sources of heterogeneity identified above and strengthen cross-study comparisons. The claim of acupoint specificity likewise merits more critical evaluation. Although some animal and clinical data support selective effects of particular acupoints (e.g., PC6 in bradycardia (107), specificity has been difficult to establish rigorously because of inadequate control points, small samples, and the lack of a biologically grounded taxonomy of acupoint indications (106). A double-dissociation framework comparing the selective effects of different acupoints on distinct outcome domains, proposed for testing acupoint functional specificity (110), offers a more rigorous design for determining whether effects are acupoint-specific or reflect general neuromodulation. Until such evidence accumulates, acupoint specificity should be regarded as plausible but not definitively established.

### 7.2 The animal-to-human translational gap

Animal experiments have revealed the molecular mechanisms of EA-mediated lipid regulation and cardiovascular protection, but translating these preclinical findings to clinical practice remains challenging because of fundamental differences between animal models and human pathophysiology. For example, in APP/PS1 double-transgenic Alzheimer's disease mice, EA reduced amyloid plaque deposition by increasing IDE expression (111), and in rat CIRI models, EA upregulated PI3 K/Akt pathway factors (81). These rodent-based findings lack direct human evidence, and no adequately powered, sham-controlled multicenter randomized controlled trial has yet confirmed the clinical efficacy of EA in cardiovascular diseases such as hypertension (18). Even within animal models, outcomes are not always consistent: a study in an aromatase-inhibitor rat model of polycystic ovary syndrome found that acupuncture did not ameliorate the associated metabolic disturbances (112). The methodological quality of clinical studies is also variable: systematic reviews highlight small sample sizes, lack of randomized controlled designs, and high risk of bias. A meta-analysis of 2,641 parturients showed that EA improved post-cesarean pain and gastrointestinal function, but with extremely high heterogeneity (I^2^ > 90%) and highly variable acupuncture protocols (113). Restraint devices and stimulation parameters used in animal experiments (e.g., 3D-printed fixators for mice (114) cannot fully reproduce the complex neuro–endocrine–immune responses of humans. EA shows potential in long COVID-related inflammatory and metabolic disorders through the vagus-mediated cholinergic anti-inflammatory pathway (53), but mapping mechanisms from animals to humans will require further translational research, particularly the broader application of psychophysical tools such as quantitative sensory testing to clarify central mechanisms in clinical populations (115, 116).

Beyond species differences, EA responses and lipid outcomes may be modified by patient-level factors such as sex, age, comorbidities, and concomitant medications, yet the current clinical evidence does not allow these variables to be disentangled. Future trials should report or stratify these characteristics to identify which populations are most likely to benefit from EA-based lipid management.

### 7.3 Specific antagonists and the limits of causal inference

Current mechanistic studies of EA are limited in establishing causality and target specificity; most demonstrate correlational changes after EA rather than causal effects (117). For example, EA regulates inflammatory cytokines and neurotransmitters in insomniac rats (118) and acts through TRPV1 signaling (119), but without gene-knockout or specific-antagonist validation, parallel pathways cannot be excluded. The TRPV1−/− mouse is an instructive exception: EA-induced signaling changes disappeared in TRPV1-knockout mice, establishing the necessary role of TRPV1 (119). Comparable rigorous designs remain uncommon in PPAR*γ*/LXR*α* research, although the T0070907, GW9662, PPAR*γ* siRNA, and MerTK-knockout experiments discussed above provide encouraging examples. Methodologically, the double-dissociation framework has been proposed to test acupoint functional specificity by comparing the selective effects of different acupoints on different physiological outcome domains (110). In practice, however, many clinical trials lack appropriate control groups; for example, a study of the effects of EA on the electrocardiogram could not reach definitive conclusions because of the absence of a control group and the small sample size (120). The literature has been criticized for insufficient awareness of the distinction between causal treatment effects and non-causal associations, and for underusing tools such as causal directed acyclic graphs. Systematic reviews recommend future adoption of CRISPR-based microbiota editing, pharmacokinetic–pharmacodynamic modeling, and Bayesian adaptive trial designs to strengthen causal inference.

### 7.4 Potential adverse consequences of PPAR*γ*/LXR*α* activation and the therapeutic window

Although PPAR*γ*/LXR*α* activation is presented in this review as a beneficial effector of EA, a balanced interpretation requires explicit consideration of its potentially adverse consequences. Pharmacological activation of PPAR*γ* with thiazolidinediones (TZDs) promotes adipogenesis and dose-dependent weight gain and can cause fluid retention and edema through enhanced sodium reabsorption in the renal collecting duct, effects that have limited the clinical use of these agents (121). Systemic LXR*α* activation likewise raises concern: in the liver, LXR*α* directly induces the lipogenic transcription factor SREBP-1c and downstream fatty-acid-synthesis genes, promoting hepatic triglyceride synthesis and steatosis and increasing plasma triglycerides, a major obstacle to LXR agonists as systemic therapeutics (122). These on-target liabilities—weight gain and fluid retention on the PPAR*γ* side, and hepatic lipogenesis and hypertriglyceridemia on the LXR*α* side—thus define a potential therapeutic window within which receptor modulation is presumed to be safe (122). Several considerations may mitigate these risks in the EA context. First, EA engages PPAR*γ*/LXR*α* signaling in a tissue- and cell-type-restricted manner rather than through systemic pharmacological agonism, as suggested by the normalization of receptor overload in lipid-laden organs and by receptor engagement primarily in macrophages and immune cells (12, 43). Second, the magnitude of receptor modulation achieved by EA is likely to be substantially lower than that produced by high-dose pharmacological agonists, reducing the probability of reaching the threshold for adverse lipogenic or fluid-retention effects (121, 122). Nonetheless, the available studies do not directly measure whether EA-induced PPAR*γ*/LXR*α* activation could adversely affect hepatic lipid handling or body-fluid balance in vulnerable populations, and this question should be addressed in future evaluations of EA safety and dose–response. A balanced assessment of benefit and harm will therefore require tissue-specific outcome measures—hepatic triglyceride content, body weight, and fluid status—alongside the lipid-lowering and efferocytic endpoints emphasized in this review.

## 8 Future directions

### 8.1 Optogenetics and chemogenetics for dissecting acupuncture neural circuits

The studies reviewed here indicate that EA improves lipid metabolism and cardiovascular function through the PPAR*γ*/LXR*α* pathway, but the specific neural pathways involved still lack cell-type-specific causal evidence. Although pharmacological antagonists such as GW9662 and T0070907 reverse the protective effects of EA, they cannot localize the specific neuronal subpopulations that transduce the signal. Optogenetic and chemogenetic approaches may resolve the “acupoint stimulation–central integration–peripheral effect” neural circuit in space and time. Previous studies show that microinjection of a PPAR*γ* inhibitor into the RVLM completely blocks the antihypertensive effect of acupuncture, whereas an agonist mimics it, identifying central PPAR*γ* as a key node (41). Future studies could use the Cre-loxP system to generate PPAR*γ*-conditional knockout mice and, in combination with viral vector-mediated expression of light-sensitive channel proteins, specifically activate or inhibit the sensory afferents innervating ST36 and their upstream central projections. By observing the responses of the PPAR*γ*/LXR*α*/ABCA1 pathway in the liver or macrophages after manipulating specific neural circuits, the central driving mechanism of EA-mediated lipid regulation could be established. High-throughput approaches are already expanding the target landscape: Oxford Nanopore full-length transcriptomics has shown that EA ameliorates hepatic lipid metabolic disorder by suppressing hepatic Pdia3/Perk/Qrich1 expression (123), and analogous transcriptomic profiling has mapped nutrient-regulated gene programs in muscle (124). Long-term chemogenetic modulation of specific brain regions (e.g., hypothalamus or nucleus of the solitary tract), combined with lipid metabolomics (125), could further reveal how the neuro–immune network reprograms systemic lipid homeostasis through the PPAR*γ*/LXR*α* axis.

### 8.2 Toward PPAR*γ*/LXR*α*-guided standardization of EA protocols

Current clinical protocols for EA in hyperlipidemia and related cardiovascular diseases are highly heterogeneous in parameters and outcome evaluation. Multiple meta-analyses show that EA is superior to conventional drugs or behavioral therapy in lowering TG, TC, and LDL-C, but acupoint selection, frequency, and treatment course differ substantially across studies. Establishing standardized protocols on the basis of the PPAR*γ*/LXR*α* molecular target is a promising direction. First, the optimal EA parameters for activating the PPAR*γ*/LXR*α* pathway need to be defined. Animal experiments show that 2-Hz low-frequency EA at ST36 and ST40 downregulates SREBP-2 and upregulates the LDL receptor, improving the lipid profile (15), and that 30-min daily EA for 3 days in a MIRI model promotes macrophage M2 polarization through the PPAR*γ*/NF-*κ*B pathway (51). Structured protocols for evaluating the effects of EA on glucose–lipid metabolism in type 2 diabetes have likewise been proposed (126). Future multicenter randomized controlled trials should systematically compare the effects of different frequencies (e.g., 2 Hz vs. 100 Hz), waveforms, and needling durations on serum lipid profiles and on downstream LXR*α*/ABCA1 expression, in order to identify the dose–response window that maximally activates this pathway (108). Genotype-guided stratification is another avenue worth exploring. Given individual differences in the PPAR*γ*/LXR*α* pathway, detecting single-nucleotide polymorphisms such as PPAR*γ* Pro12Ala may help predict sensitivity to EA (2). For outcome evaluation, in addition to conventional lipid parameters, serum inflammatory markers (e.g., IL-10, TNF-*α*) and PPAR*γ*/LXR*α* mRNA levels in peripheral blood mononuclear cells could be included as endpoint measures, providing objective quantification of the biological effects of EA and supporting the development of mechanism-based clinical guidelines (127).

## 9 Conclusion

Current evidence constructs a coherent chain linking EA intervention to PPAR*γ*/LXR*α* pathway activation and, in turn, to improved lipid metabolism and cardiovascular protection. At the molecular level, EA modulates the expression of PPAR*γ* and LXR*α*, an essential step in its lipid-lowering action. In HFD-induced atherosclerotic models, EA activates the PPAR*γ*–LXR*α*–MerTK pathway, upregulating ABCA1, ABCG1, PPAR*γ*, LXR*α*, and MerTK in myocardial macrophages, enhancing efferocytosis, and reversing lipid deposition; PPAR*γ* knockdown abolishes these protective effects (12). In CIRI models, EA at LI11 and ST36 increases PPAR*γ* expression and suppresses oxidative stress and inflammation, and the PPAR*γ* antagonist T0070907 reverses this protection (39). EA also indirectly activates the PPAR*γ*/LXR*α* axis through upstream signals such as AMPK and SIRT1, promoting the expression of the reverse-cholesterol-transport genes ABCA1 and ABCG1. In preclinical models, EA lowers serum TC, TG, and LDL-C, and improves hepatic steatosis and atherosclerotic plaque burden, phenotypic improvements that are associated with PPAR*γ*/LXR*α* pathway activation. Elucidating the molecular mechanism of EA-mediated lipid regulation—particularly the role of the PPAR*γ*/LXR*α* pathway—provides a biological basis for the modernization and international acceptance of acupuncture. Integrating traditional acupuncture efficacy with modern molecular targets helps dispel the “black box” and strengthens the recognition of acupuncture as a scientific intervention. Multiple randomized controlled trials and meta-analyses have confirmed the clinical efficacy of acupuncture in NAFLD, hyperlipidemia, and insulin resistance, yet the lack of mechanistic explanation has limited its recommendation level in mainstream guidelines. By defining the specific pathways through which EA regulates inflammatory factors and lipid-metabolic enzymes via the PPAR*γ*/LXR*α* axis, this review provides quantifiable and testable scientific evidence for acupuncture in metabolic disease. Mechanistic insight also guides protocol optimization: because different frequencies and waveforms may differentially affect PPAR*γ* expression, a deeper understanding of this mechanism can support standardized EA parameters in clinical practice. Finally, PPAR*γ* and LXR*α* are classic drug-development targets (128); the fact that EA activates them suggests that acupuncture mayachieve metabolic effects that functionally resemble pharmacological receptor activation, although this analogy rather than direct receptor–ligand binding remains to be validated experimentally, providing a theoretical foundation for incorporating acupuncture into the cardiovascular-disease prevention framework as an alternative or complementary therapy.
